# The Estrogen Hypothesis of Obesity

**DOI:** 10.1371/journal.pone.0099776

**Published:** 2014-06-10

**Authors:** James P. Grantham, Maciej Henneberg

**Affiliations:** School of Medical Sciences, The University of Adelaide, Adelaide, Australia; University of Missouri, United States of America

## Abstract

The explanation of obesity as a simple result of positive energy balance fails to account for the scope of variable responses to diets and lifestyles. It is postulated that individual physiological and anatomical variation may be responsible for developing obesity. Girls in poor families develop greater adiposity than their male siblings, a trend not present in richer environments. This indicates strong influence of estrogen on fat accumulation irrespective of poor socioeconomic conditions. Obesity rates in males and females of developed nations are similar, while in poorer nations obesity is much more prevalent in females. Female to male ratio of obesity correlates inversely with gross domestic product. Therefore, the parity of male and female obesity in developed countries may result from male exposure to environmental estrogen-like substances associated with affluence. These hormonally driven mechanisms may be equally active within both sexes in more developed areas, thereby increasing overall obesity.

## Introduction

The accretion of overweight and obese individuals is becoming a universally defining feature of twenty-first century. Long considered a burden of Western nations, the improving conditions of developing nations have rendered them vulnerable too. It is currently estimated that 1.4 billion individuals are overweight and 500 million people are obese globally, many of them in developing nations [1]. This trend appears as if it will perpetuate indefinitely into our future, with childhood obesity becoming increasingly prominent [2].

Obesity is no longer endemic to any particular group, carving its way through society and societies. In developed nations, there appears to be no marked sexual dimorphism of adiposity. In the United States, amongst those of European heritage, males have reported obesity rates of 27.5% compared to 24.5% for women [3]. In 2009, England reported 24% of women as obese and 22% of men [4]. This relative parity is consistent amongst developed nations. Congruently, the most widely known scale for adjudging corpulence, BMI, is calculated using precisely the same methods for men and women, inferring that the impact of sex is negligible [5]. This practice fails to consider the strikingly different basic body compositions of men and women.

Interestingly, amongst developing nations, obesity is much more prominent amongst women. The obesity rates in the largest developing nations reflect this trend. Obesity is more than twice as common amongst women in India (2.8% vs 1.3%) and a similar trend is exhibited in China where just 2.4% of men are obese compared to 3.4% of women [6]–[7]. Globally, 14% of women are classified as obese, a figure almost 50% higher than their male counterparts at 10% [8]. The magnitude of this contrast is slightly ameliorated by the confounding equitable obesity rates between men and women in countries such as the United States and most of Europe [7]. Amongst many of these nations, dietary composition and caloric consumption are strikingly inimical to weight gain [9]. Therefore, it is in these environments that the traditional conceptualisations of the energy input, energy output relationship begin to falter. This highlights the importance of hormonal influences in weight regulation, with examples of obese individuals in environments unlikely to deliver an external positive energy balance.

The observed discrepancy between men and women in such conditions suggests variable responses along the sex divide. If this fact is accepted, the next step is to determine the potential mechanisms behind such a difference. As with determining the cause of any discrepancy between the sexes, hormonal influences come to the fore of the discussion. It is not within the scope of this study to elucidate the precise hormonal mechanisms through which the aforementioned trends develop. Instead, the focus will be to establish the trend and briefly theorise the most likely aetiologies.

## Materials and Methods

There were several sources of data used in the formation of our paper. Data were collected between 1986 and 1995 from intentionally distinctively varying socioeconomic groups within South Africa (Cape Coloured population) using known markers of community affluence. Two districts were chosen, the affluent, urbanised Greater Cape Town and the poorest, rural region of Klein Karoo. The most prestigious schools within the Greater Cape Town region were then targeted for the study as were the most underprivileged schools within the Klein Karoo area. The ethics of the study were approved by the appropriate committees with permission and collaboration coming from school authorities. Initially 906 boys and 1068 girls aged between 5 and 20 were cross-sectionally examined from the metropolitan district as were 834 boys and 930 girls from the rural outposts. Various anthropometric measurements were obtained for each child with particular emphasis on skin fold testing. Exercise capacity and neuromuscular reaction times were also recorded and routine blood analysis was performed. All participants were categorised into one of five socio-economic classes based on parental mode of employment. Results were broadly grouped into four separate categories, urban boys, urban girls, rural boys and rural girls. The average results for each variable were scrutinised and contrasted. Information regarding the population studied and exact recording procedures are outlined in the original paper [10]. In order to illustrate the overall observable trends, the average sum of three skin-fold measurements (triceps, subscapular and abdominal) were calculated for each age group for all four of the major groups outlined above. These were then recorded and graphed together in order to illustrate the changes across each sample.

In an attempt to ameliorate the influence of environmental factors, the weight trends of several siblings were analysed. Additionally, to highlight the importance of sexual dimorphism within challenging climates, only rural individuals were selected. Manual processing was then performed to select for individuals almost certain to be siblings based on stringent criteria. Only two individuals with the same surname, born more than eighteen months apart, living in the same place (attending the same school) and possessing the same parental socio-economic status were selected. Following this, only eight pairs of brother-sister combinations were incorporated into the graph for the purposes of illustrating the sex based discrepancy that develops with age in the same families, most likely sharing food and living arrangements.

To demonstrate the pattern of weight gain amongst individuals with extreme cases of obesity, the fattest five individuals from each group were automatically selected, based on abdominal skin-fold recordings. The individuals selected were those with the five highest unique recordings at some point during the cross-sectional measurements. This process was conducted independently for urban males, urban females, rural males and rural females respectively. Following this, the results were combined and incorporated into a single figure for illustrative purposes.

Data on all nations of the world were also used here. The Gross Domestic Product was obtained from the United Nations for each available nation [11]. These values were then considered against the male to female obesity ratio of obesity calculated from WHO files. The ratio was obtained by dividing the male prevalence of obesity by the female prevalence of obesity, expressed as percentages of the adult population. This figure is an attempt to illustrate the changing features of obesity with respect to improving environmental conditions. This result was also subsequently represented in tabulated form with the mean national prevalence of obesity contrasted with the gross domestic product (GDP) of each nation stratified above and below $13,000(U.S). All data were stored within Microsoft Excel with the statistical analysis conducted through the SPSS Statistics v 19.00 program. Polynomial and exponential regression lines were fitted to the scattergrams of skinfold thicknesses of children and adolescents, linear regression was used to explore the relationship between GDP per capita and ratios of male to female obese persons. Significance of correlation coefficients was assessed by observing whether their confidence intervals include zero or not. Frequencies of male and female persons with BMI>30 kg/m^2^ were used to construct these ratios. SPSS calculated averages were compared by this program's t-tests for independent samples.

## Results

**Figure 1:**
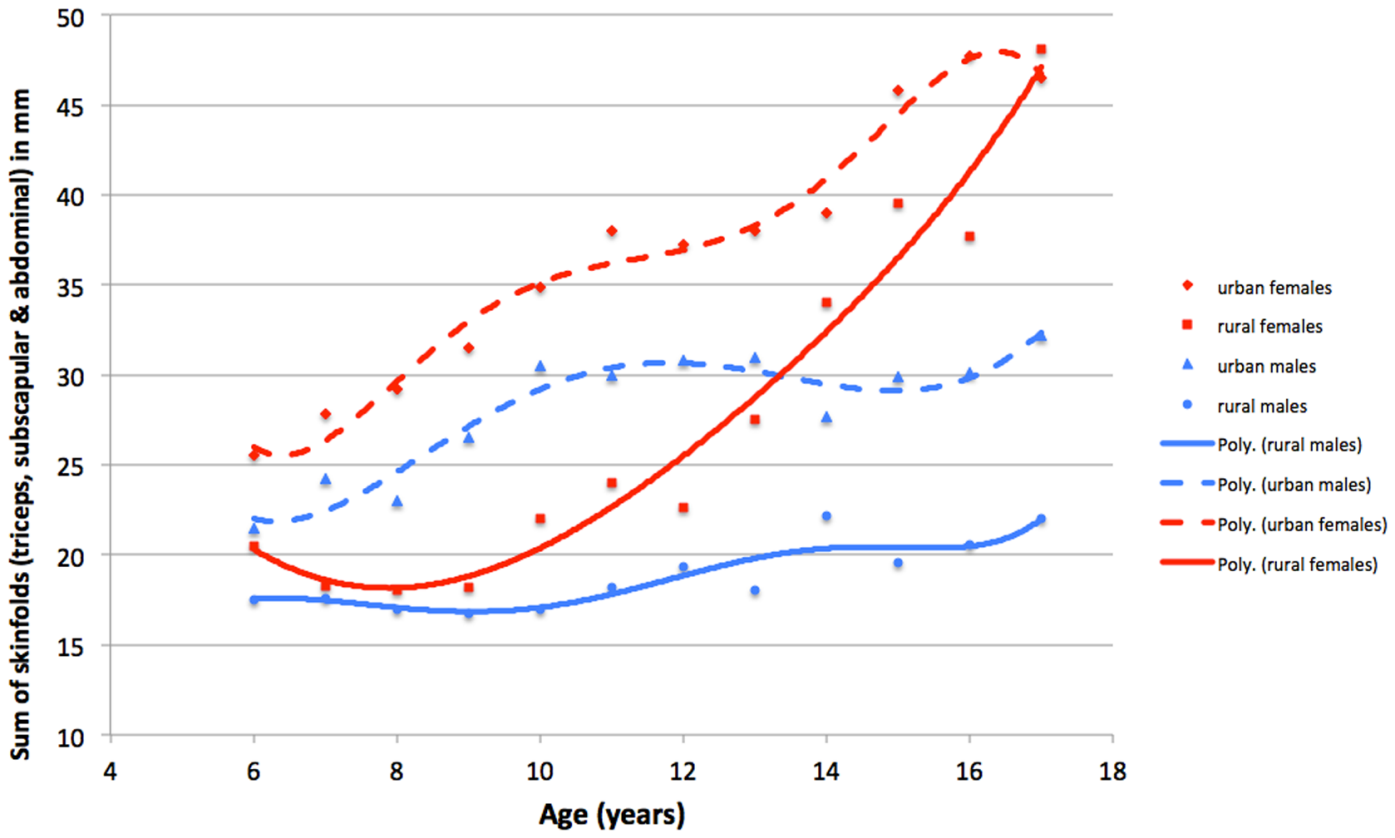
The average sum of skin-folds across age for all four groups based on sex and urban-rural divide.

As demonstrated by **Figure 1**, diverse trends exist in the average skin-fold recordings until adulthood for both sexes. Notably, the thinnest group is the rural male cohort, with significantly (t-tests, p>0.05) higher recordings taken from their urban brethren. Perhaps more interestingly, the rural girls demonstrate substantially lesser pre-menarcheal skin-folds recordings than both male and female city dwellers. There is also a delayed menarche within the rural sample of girls, owing to nutritional scarcity [12]–[15]. However, post-menarche there is a sharp increase in the adiposity of this cohort and recordings reach parity (no statistically significant difference) with the urban girls by the age of 17. This parity is not observed between the rural and urban boys, while amongst girls the inherited mechanisms take over the environmental differences.

**Figure 2:**
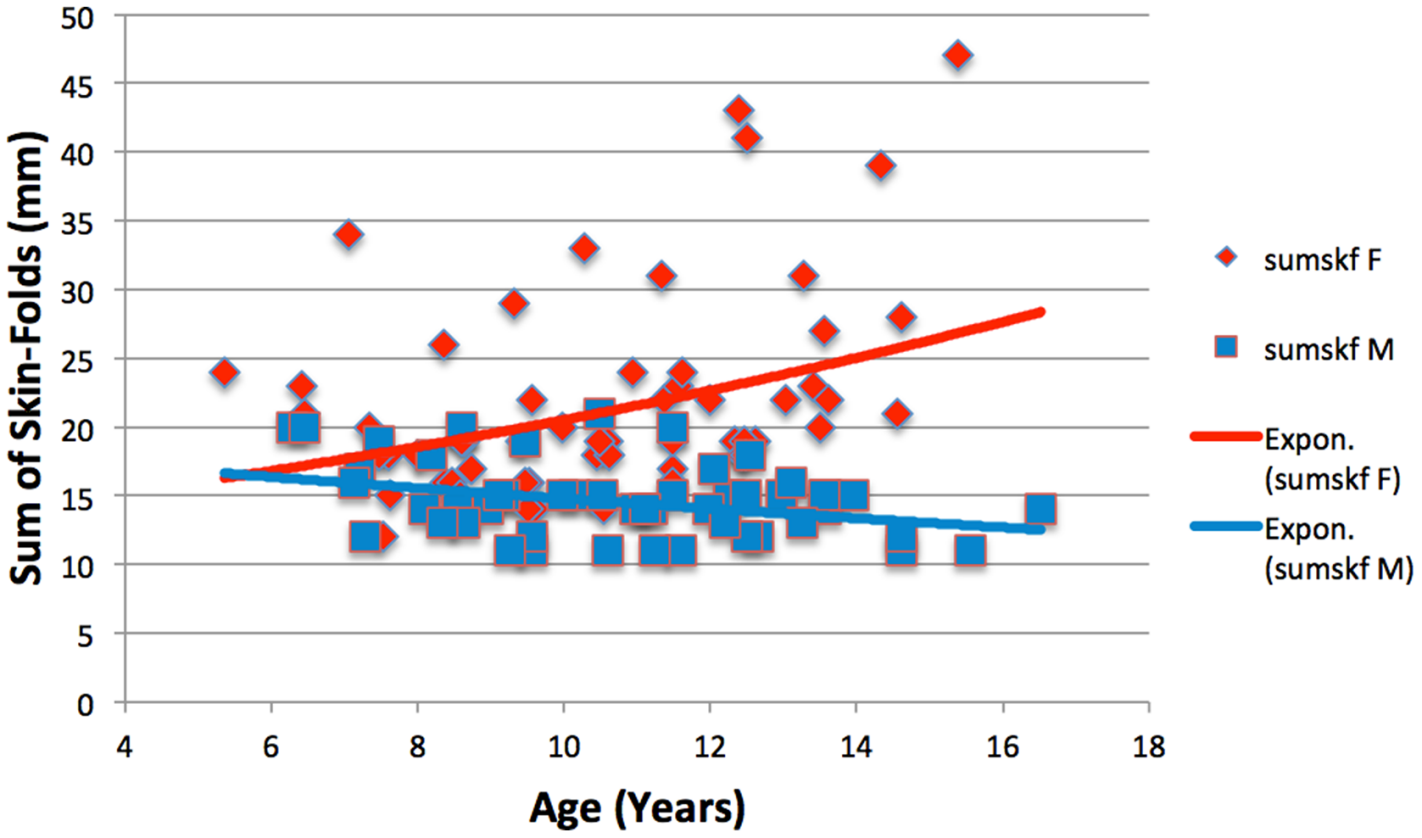
The development of skin-fold recordings with age amongst sets of brother-sister siblings in a rural environment.

The relatively higher fatness gain observed amongst female participants, irrespective of nutritional access is further illustrated in **Figure 2**. The mixed-longitudinal recordings were taken from 8 rural, brother-sister pairs where each individual was measured 6–8 times at yearly intervals. By matching only these genetically similar pairs, it is possible to ameliorate any autosomal genetic influence. Additionally, these paired siblings, living in the same households, would likely experience similar dietary intake and lifestyles. Therefore, this sample best demonstrates the sex driven adiposity discrepancies between males and females in an environment of limited food access. As can be seen, a significant increase (r = 0.40, p<0.01) is noted amongst the female sample, owing to hormonally driven factors. Coevally, the trend observed amongst the male participants (r = −0.34, p<0.05) reveals a decline in skin-fold recordings, a pattern more in line with the expected weight trends of such an environment. Although details of living circumstances of each individual child are unknown, the overall characteristics of living conditions in rural settlements of “Cape Coloured” people under apartheid are aptly characterised by the observation that at the commencement of the collection of longitudinal observations the average income per capita in those areas was US$10.0 per month [10].

**Figure 3:**
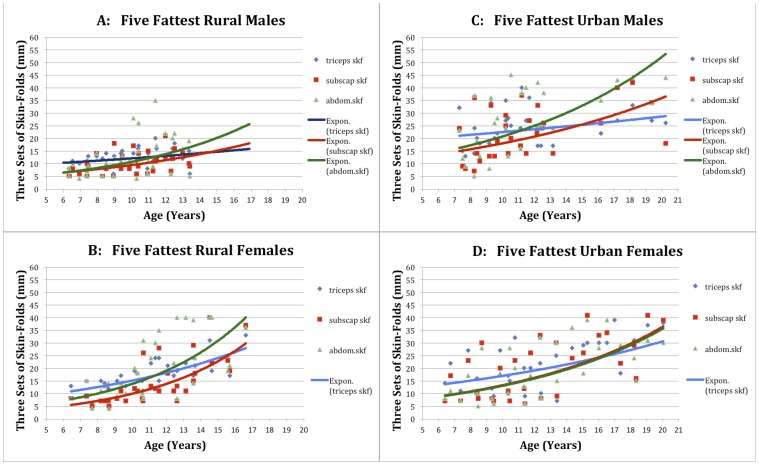
Longitudinal skinfold recordings for the five fattest individuals among: A – rural males, B, rural females, C- urban males, D- urban females.

Finally, Figures 3A-D illustrate the sum of skin-folds of male and female individuals, ranging from the age of 7 to 19 years. The samples were taken from the disadvantaged, rural area of Klein Karoo and the more affluent Greater Cape town urban area [10]. Participants were selected based on their abdominal skin fold recordings in order to attain only the five most corpulent individuals for each of the four cohorts (Rural male, rural female, urban male and urban female). The overall trends witnessed in the four graphs are relatively consistent with those expected in light of the averages. As expected, the least fat of the top five are found amongst the rural male cohort **(** **Fig.3A** **)** with relatively modest increases in adiposity across the teenage years. Some irregularities did, however, develop. It was found that the fattest of the urban males **(** **Fig.3C** **)** had comparable skin-folds with each of the female cohorts, despite average skin-fold recordings being significantly (p<0.05) lower in all urban males than in urban females. Another noteworthy trend that developed was the conformity of results between the two female cohorts by age 17, consistent with previously mentioned observations. The five fattest urban females **(** **Fig.3D** **)** showed a relatively steady fat gain across the formative years with the trend-lines almost reminiscent of a linear pattern. Contrastingly, the rural females **(** **Fig.3B** **)** demonstrated relatively meagre fatness initially, followed by a rapid acceleration at approximately age 12. This timeframe is consistent with the well-documented pre and peri-menarche weight gain observed amongst teenage women due to estrogenic exposure [16]. The significance of recording such a phenomenon in the context of this paper resides in the fact that both sexes experience precisely the same environment in terms of diet and socioeconomic status as they are members of same families. The rural disadvantaged population has a particularly low caloric intake as evidenced by low values of skinfold thicknesses. Therefore, this sex driven pattern of weight gain demonstrates the importance of metabolic and hormonal factors, independent of energy consumption.

**Figure 4:**
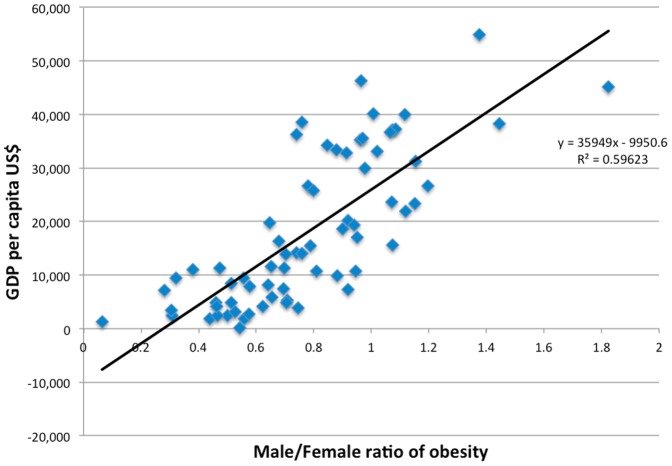
Relationship between GDP and the Male/Female Ratio of Obesity.

Figure 4 depicts the relationship between national Gross Domestic Product and the male to female ratio of obesity. As can be seen from the graph, the correlation is highly statistically significant (r = 0.77, p<0.01), despite the data arising from all regions of the globe being subject to possible reporting inaccuracies. This finding is consistent with the assertion that male and female obesity prevalence approaches parody with improving living conditions. To highlight this relationship is important as, the trend associated with Gross Domestic Product may be attributable to a number of factors associated with affluence. Some of these include nutritional abundance and dietary composition, reduced physical demands and environmental contaminants associated with industry.

The dichotomy of obesity trends is represented in Table 1. The average male and female obesity prevalence is documented, with nations categorised based on Gross Domestic Product. Nations with a Gross Domestic Product less than thirteen thousand American dollars per annum demonstrated significantly lower rates of male obesity (p<0.001) than amongst nations that exceed this threshold. This is in direct contrast to the patterns observed amongst women, with practically no difference documented between rich and poor countries. A second observation can also be drawn from the table. Within affluent countries, the prevalence of male and female obesity is comparable; the difference between means is not statistically significant. Conversely, throughout nations with modest wealth, almost twice the prevalence of female obesity is noted in comparison to their male counterparts and this difference is significant (p<0.02).

**Table 1:** Average Levels of Obesity (%) Amongst Males and Females in Rich and Poor Countries.

Gross Domestic Product ($US Per Year)	Sample Size	Males Obesity Prevalence (Standard Deviation)	Female Obesity Prevalence (Standard Deviation)
≥13000	36	16.4 (7.0)	17.3 (8.4)
<13000	33	9.8 (8.7)	16.7 (12.4)
T Value		3.5	0.3
P Value		0.001	0.797

## Discussion

### Quality and Reliability of Results

In terms of qualitatively analysing the validity of the results, it is necessary to evaluate each of the used data sets independently. The data regarding the African children are quite scrupulous. One contention may be found in selecting a small sample size to illustrate the contrasting patterns of weight gain for the fattest five individuals from each group. However, the four samples were chosen indiscriminately based on the same criteria. Moreover, the selection of a small sample was intended for the purposes of illustrating an idea through an aesthetically pleasing microcosm of the larger sample. In essence, the demonstrated relationship shares a strong fidelity with the overall trend within all Cape Coloured children.

In the instance of the relationship between GDP and the obesity ratio, no manipulation of the data was conducted. However, the information was compiled by the authorities based on national self-reporting. This means that a certain amount of scepticism must be exercised when considering the validity of the results. Having said this, these are the most accurate data available on the matter.

### Hypothesis

The findings of this paper suggest that hormonally driven weight gain occurs more significantly in females than males. This is evidenced by the fact that average prevalence of obesity in males differs highly significantly between rich and poor countries whereas there is no significant difference between those countries in the average prevalence of female obesity. In light of this information, it may be appropriate to suggest that men appear to be more accurate barometers of the nutritional state of the community. This is supported by the significant correlation established between national gross domestic product and the male to female ratio of obesity. As caloric intake is generally correlated with Gross Domestic Product, it suggests there are proportionately greater levels of male obesity in nations with superfluous dietary intake.

With this in mind, it is important to consider why women demonstrate greater consistency across the world with regard to obesity rates. Amongst women, oestrogen exposure is known to cause weight gain, primarily through thyroid inhibition and modulation of the hypothalamus [17]–[18]. It is suggested that this is a mechanism by which they may accrue adequate fat reserves to sustain a pregnancy [19]. In the environment of evolutionary adaptation, food resources were relatively limited. In such a situation, the ability of oestrogen to promote weight gain based on finite food resources probably engendered a significant selective advantage as it would have augmented the period in which a successful pregnancy was viable. The resultant outcome is that women respond to barely sufficient caloric consumption vastly different from men, with fat depots more readily preserved and developed. This trend is consistent with the dichotomy of obesity rates between men and women in developing nations, where nutritional access is often impaired.

The question thus turns to our modern Western society and why this process is not as coarsely observable, with the rates approaching parody as national gross domestic product increases. The answer lies in the superfluous nature of the modern Western diet. The mechanism of adipose preservation through oestrogen is ultimately unnecessary in such an environment and may, in fact, become deleterious. Therefore, very few individuals demonstrate the trends of slimness that would have been commonplace in the past and still are today in developing nations.

Aside from a simple matter of caloric intake, there appears to be other characters in the story of weight gain. The influence of soy on contributing to weight gain has been recently established [20]. Authors of this finding have cited xenoestrogens contained within soy products as a likely culprit [20]. Perhaps, in societies with particularly high dietary saturation of soy, this works to “feminise” the males. If such a fact was substantiated, the soy exposure would allow men in those communities to artificially imitate the female pattern of weight gain. Interestingly, the United States, the largest consumer of soy products per capita in the entire world, belong to a select group in which male obesity rates outstrip those of their female counterparts [3], [21]. Another well-established source of xenoestrogen is polyvinyl chloride, known as PVC [22]. This product is in prominent use in most wealthy countries with widespread applications, from plastic medical devices to irrigative piping [23]–[24]. The use of plastics is known to correlate highly with the Gross Domestic Product of a nation [25]. Therefore, it stands to reason, given the trends of weight gain observed, that this may play a role.

Another possible contributing factor could be relaxed natural selection acting in those countries with the sizeable Gross Domestic Products. There are numerous accounts of micro-evolutionary changes occurring within just two generations [26]–[28]. In these nations, perhaps the ameliorated physical demand of the modern lifestyle has led to micro-evolutionary changes in the levels of testosterone and oestrogen amongst men. This would certainly explain the various concerns about sperm count reductions in many nations [29]. As it stands currently, these explanations are simply hypotheses, but it seems apparent that hormonal concentrations could lie at the heart of this issue. It is entirely possible that, other unconsidered mechanisms are also playing an integral role in equalising obesity rates between men and women.

### Detracting Arguments

One contention regarding the study is that not all of the sex discrepancy is attributable to physiological mechanisms. It is well established that women are more susceptible to many endocrine diseases, leading to weight gain, including hypothyroidism and Cushing's syndrome [30]–[31]. It is estimated that women are two to eight-fold more likely to experience hypothyroidism, with Cushing's syndrome also five times more prevalent amongst women [32]–[33]. Hypothyroidism is particularly common, a study conducted late last century showed 0.3% of the United States population experience overt hypothyroidism but a further 4.3% have subclinical hypothyroidism [34]. This remarkable pathological dimorphism may contribute to the discrepancy observed between male and female obesity rates worldwide. However, it is likely that this only explains a minor element of the sex driven variance because subclinical hypothyroidism has limited effects on inducing weight gain [35].

The other issue that may be raised lies in the knowledge of the contrast in natural body fat composition that exists between men and women. In 2009, the average adult male in the United States was composed of approximately 21% body fat compared with the average female, with a body fat of around 28% [36]. The discrepancy is also present amongst professional athletes with most recorded body fat percentages ranging between 14% and 20% for women and between 6% and 13% amongst men [36]. Additionally, it is known that women have thicker subcutaneous fat deposits [37]. Therefore, a reasonable conclusion would be to assume that irrespective of actual body weight relative to health status, skin-fold recordings will be higher amongst females than males. This may be true but obtaining skin-fold measurements is widely accepted as the best field-based method of representing body fatness, irrespective of sex.

### Implications

Our hypothesis may have widespread implications pending further substantiation. First and foremost, it may inform those combating the obesity epidemic about the importance of hormonal regulation variation. Currently, the influence of oestrogen is often underappreciated in the development of obesity with the same recommendations of healthy weight ranges standing for both women and men [5]. In future, perhaps it is appropriate to introduce a sex based correction to equalise weight classifications such as BMI, particularly in countries without an abundant food supply.

Additionally, this paper once again raises some previously considered issues, namely the impact of certain substances on our biology. The issue of soy and polyvinyl chloride is a contentious one. Both are known to contain xenoestrogens which may contaminate our diet and our environment. The World Health Organisation is sufficiently concerned about the impact of PVC as to commission numerous reports on the matter [38]. This implies that a leading international health body is conscious of the growing issue of xenoestrogen contamination and cognisant of the potential ramifications.

Given the weight of scientific opposition to many of these products, prudence would dictate that further consideration should be encouraged. This cautious approach must at least be extended to the many applications of these products containing xenoestrogen that come into significant contact with food supplies and water reservoirs. Some of these applications include vast piping networks, composed of PVC and the plethora of consumable products in which soybean derived products are heavily incorporated, including vegetable oil, and various milks and cheeses [39]–[40]. In essence, pending further investigation, it may prove appropriate to reduce or eliminate any application with a preponderance to incorporate artificial xenoestrogens into the human biome.

With respect to background xenoestrogen saturation, numerous ethical and environmental considerations also present which are well beyond the scope of this paper. Therefore, in the interests of parsimony, these will not be elaborated on further in this text. Suffice it to say for the purposes of this paper that the apparent feminisation of men by these environmental contaminants appears to exist. The inference of such a trend is that xenoestrogen contamination may play a pivotal role in the development of obesity across many nations, particularly amongst the male population.
